# How Well Do Discharge Diagnoses Identify Hospitalised Patients with Community-Acquired Infections? – A Validation Study

**DOI:** 10.1371/journal.pone.0092891

**Published:** 2014-03-24

**Authors:** Daniel Pilsgaard Henriksen, Stig Lønberg Nielsen, Christian Borbjerg Laursen, Jesper Hallas, Court Pedersen, Annmarie Touborg Lassen

**Affiliations:** 1 Department of Emergency Medicine, Odense University Hospital, Odense, Denmark; 2 Department of Infectious Diseases, Odense University Hospital, Odense, Denmark; 3 Department of Respiratory Medicine, Odense University Hospital, Odense, Denmark; 4 Department of Clinical Pharmacology, University of Southern Denmark, Odense, Denmark; Universidade do Extremo Sul Catarinense, Brazil

## Abstract

**Background:**

Credible measures of disease incidence, trends and mortality can be obtained through surveillance using manual chart review, but this is both time-consuming and expensive. ICD-10 discharge diagnoses are used as surrogate markers of infection, but knowledge on the validity of infections in general is sparse. The aim of the study was to determine how well ICD-10 discharge diagnoses identify patients with community-acquired infections in a medical emergency department (ED), overall and related to sites of infection and patient characteristics.

**Methods:**

We manually reviewed 5977 patients admitted to a medical ED in a one-year period (September 2010-August 2011), to establish if they were hospitalised with community-acquired infection. Using the manual review as gold standard, we calculated the sensitivity, specificity, predictive values, and likelihood ratios of discharge diagnoses indicating infection.

**Results:**

Two thousand five hundred eleven patients were identified with community-acquired infection according to chart review (42.0%, 95% confidence interval [95%CI]: 40.8–43.3%) compared to 2550 patients identified by ICD-10 diagnoses (42.8%, 95%CI: 41.6–44.1%). Sensitivity of the ICD-10 diagnoses was 79.9% (95%CI: 78.1–81.3%), specificity 83.9% (95%CI: 82.6–85.1%), positive likelihood ratio 4.95 (95%CI: 4.58–5.36) and negative likelihood ratio 0.24 (95%CI: 0.22–0.26). The two most common sites of infection, the lower respiratory tract and urinary tract, had positive likelihood ratios of 8.3 (95%CI: 7.5–9.2) and 11.3 (95%CI: 10.2–12.9) respectively. We identified significant variation in diagnostic validity related to age, comorbidity and disease severity.

**Conclusion:**

ICD-10 discharge diagnoses identify specific sites of infection with a high degree of validity, but only a moderate degree when identifying infections in general.

## Introduction

Credible measures of disease incidence, trends and mortality are critical for a proper public healthcare management. This information can be obtained through surveillance using manual chart review, but this is both time-consuming and expensive [1]. Surveillance of infections often depends on notifications from the physicians. However, because patients are registered with diagnose codes at their discharge or transfer from department to department, it is possible to use discharge diagnoses as surrogate markers of infection. Studies examining the validity of discharge diagnoses identifying infections have previously to a large extent only focused on specific sites of infection, with varying results. The validity depends on which infection the patient presents with, the patient population, and setting examined [2].

Only a few studies have assessed the validity of ICD-10 codes for infections in general [3]–[5], and it is unknown if the validity changes in specific patient subgroups.

The aims of this study were to determine, to which degree discharge diagnoses of infection could accurately identify community-acquired infections in an emergency department (ED) setting; and to assess if the sites of infection, baseline patient characteristics and disease severity affect the validity of the discharge diagnoses.

## Materials and Methods

We conducted a cross sectional study of all patients admitted to the medical ED at Odense University Hospital, Denmark from 1 September 2010- 31 August 2011. All subjects were manually reviewed with respect to the presence of an infection at admission. We ascertained the ability of discharge diagnoses to identify the infections found by a structured manual review.

### Ethics statement

In compliance with Danish law, the study was notified to and approved by the Danish Data Protection Agency (J No 2008-58-0035), and the access to patient clinical records was approved by the Danish National Board of Health (J No 3-3013-35). No further ethical approval, or consent from participants, is needed for register-based studies in Denmark. Data were anonymised and de-identified prior data analysis.

### Study design and setting

The medical ED serves a population of 235,000 adults and serves as a medical admission unit for the following medical specialities: general internal medicine, infectious diseases, gastrointestinal medicine, geriatric medicine, rheumatology, endocrinology and respiratory medicine. The medical ED received all acutely admitted medical patients referred from either a primary care physician or from the open general ED where an emergency care physician found the patient in need of admission. At arrival, all patients had their vital signs registered and blood drawn for laboratory analysis, as a part of the clinical routine.

### Participants

Patients eligible for the study were adults (≥15 years of age) with a first time admission to the medical ED within the study period. Patients without a Danish civil registration number and patients discharged from a hospital up to 7 days prior to inclusion were excluded from further analysis.

We used a structured protocol to collect data regarding the presence of infections, based on The National Healthcare Safety Network criteria in combination with a predefined definition, where the site of infection was clinically evident [6] (Appendix S1). A manual chart review was conducted of all patients admitted to the medical ED within the study period. Physicians notes, nurses charts, data on microbiological cultures, biochemical data and radiographic imaging were reviewed, and infections identified during the first 48 hours of the admission were included. The manual chart review was done by an experienced clinical physician (DPH). If a patient had more than one site of infection associated to the given admission, we included all, and did not prioritise between them.

### Validation of chart review

We assessed the inter-observer reliability by analysing 2.5% randomly selected patients from all admissions to the medical emergency ward within the inclusion period, to examine the reproducibility of identifying infection by manual chart review. The review was done by two experienced clinical physicians (DPH and CBL), blinded to each other and the others verdict. The general inter-rater agreement, regarding the presence of all infections, was 84.1% with a kappa value of 0.68, producing a substantial strength of agreement [7]. When restricting to specific sites of infection, the inter-rater agreement was between 92.7% (lower respiratory tract) to 100% (cardiovascular).

### Data sources

#### Database

Trained data abstractors extracted and validated clinical details and vital signs at the time of admission from the electronic patient journal.

Using the unique Danish personal identification number [8], supplementary information on included patients were retrieved and linked from several large population-based registers.

#### Funen Patient Administrative System

The register comprises all hospitalisations at Odense University Hospital registered since 1974, and was used to identify all patients admitted to the medical ED within the study period, as well as the registered time of admission.

#### Danish National Patient Register

The register contains data on admission and discharge dates as well as discharge diagnosis for all patients hospitalised in Denmark since 1977, classified according to the International Classification of Diseases, 10th revision (ICD-10) from 1994 and onward [9]. For the included patients we extracted discharge diagnoses from the previous 10 years to generate a Charlson comorbidity score and grouped it to form the Charlson Comorbidity Index for each patient enrolled in the study, as a marker for comorbid illness [10].

#### Other registers and databases

Data were supplemented by information from Odense Pharmacoepidemiological Database, the laboratory information system at Department of Clinical Microbiology at Odense University Hospital, the Danish National Cancer Register, as well as the Danish National Alcohol- and Drug Treatment Register, with the aim to identify patients with immunosuppression, community-acquired bacteremia and alcoholism-related conditions [11], [12]. Data on birth, deaths and migration status were obtained from the Civil Registration System in Denmark [13].

### Definitions

In order to categorise the discharge diagnoses into sites of infection, we reviewed all ICD-10 diagnoses aggregated from the entire admission of all patients admitted to the medical ED within the study period. The ICD-10 diagnoses indicating the presence of infection are presented in Appendix S2. If a discharge diagnosis was associated with a specific microbe (e.g. A490 Staphylococcal infection, unspecified site) and did not have a specific organ relation, we classified it as the presence of infection and unknown site of infection. For additional definitions on immunosuppression, alcoholism-related conditions, organ dysfunction, comorbidity and systemic inflammatory response syndrome, see Appendix S3.

### Analysis

We assessed the validity of discharge diagnoses indicating infections in general presented as a crude value (infection yes/no) as well as stratified into sites of infection, using sensitivity, specificity, likelihood ratios and predictive values.

The diagnosis of infection was extracted from chart review and compared to the discharge diagnoses. The chart review was considered as gold standard. The distribution of these two different approaches, as well as tentative diagnoses from the medical ED, was illustrated in an area proportional Euler Diagram [14].

Baseline patient characteristics were presented as the proportion of all eligible patients, and the proportion of patients with infection according to chart review and ICD-10 discharge diagnoses.

Positive likelihood ratio was defined as the probability of a patient with infection, who had a discharge diagnosis indicating infection, divided by the probability of a patient without infection, but with a discharge diagnosis indicating infection (sensitivity/[1- specificity]). Negative likelihood ratio was defined as the probability of a patient with infection, but without a discharge diagnosis indicating infection, divided by the probability of a patient without infection and without a discharge diagnoses indicating infection ([1- sensitivity]/specificity).

95% confidence intervals were calculated for predictive values, likelihood ratios, sensitivity and specificity analysis assuming normal approximation of the binomial distribution.

Statistical analysis was performed with Stata version 13.0 (Stata Corporation, Texas, USA).

## Results

### Participants

**Figure 1:**
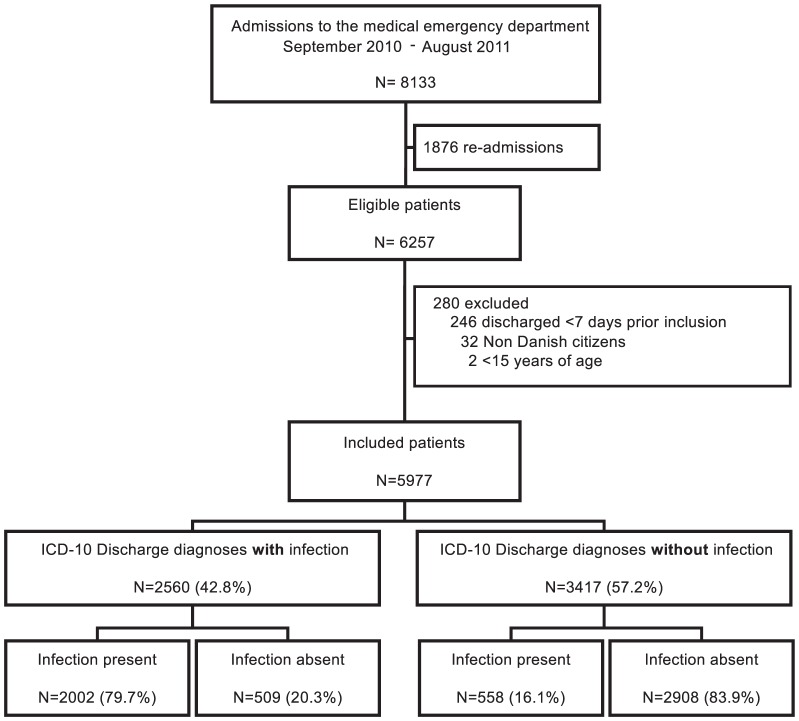
Flow chart of the study population.

A total of 6257 patients had one or more admissions to the medical ED during the study period. 280 were excluded, 5977 were included (Figure 1). The median age of the included patients was 66 years (5–95% range: 21–91 years). 2722 (45.5%) were males, and 2002 (33.5%) presented with a Charlson Comorbidity Index >2 (Table 1).

**Table 1:** Baseline characteristics in patients admitted to the medical emergency department and proportions of patients with infection identified by chart review and ICD-10 discharge diagnoses.

		Included patients (n/N)	Infection by chart review (n/N) [955CI]^1^	Infection by ICD-10 discharge diagnoses (n/N) [95%CI]^1^
N (%)		100.0 (5977/5977)	42.0 (2511/5977) [40.8–43.3]	42.8 (2560/5977) [41.6–44.1]
Gender (%)	Female	54.5 (3255/5977)	40.9 (1331/3255) [39.2–42.6]	42.5 (1382/3255) [40.8–44.2]
	Male	45.5 (2722/5977)	43.4 (1180/2722) [41.5–45.2]	43.3 (1178/2722) [41.4–45.2]
Age-categories, years (%)	15–39	19.7 (1178/5977)	31.7 (373/1178) [29.0–34.4]	29.6 (349/1178) [27.0–32.3]
	40–64	28.7 (1713/5977)	36.9 (632/1713) [34.6–39.2]	36.8 (630/1713) [34.5–39.1]
	65–84	37.8 (2257/5977)	48.6 (1098/2257) [46.6–50.7]	50.0 (1128/2257) [47.9–52.1]
	85+	13.9 (829/5977)	49.2 (408/829) [45.8–52.7]	54.6 (453/829) [51.2–58.1]
Charlson Comorbidity index (%)	0	44.1 (2637/5977)	35.9 (948/2637) [34.1–37.8]	36.9 (973/2637) [35.1–38.8]
	1–2	22.4 (1338/5977)	43.1 (577/1338) [40.5–45.8]	43.8 (586/1338) [41.1–46.5]
	>2	33.5 (2002/5977)	49.3 (986/2002) [47.0–51.5]	50.0 (1001/2002) [47.8–52.2]
Immunosupression (%)	No	86.4 (5162/5977)	39.9 (2062/5162) [38.6–41.3]	41.7 (2150/5162) [40.3–43.0]
	Yes	13.6 (815/5977)	55.1 (449/815) [51.6–58.5]	50.3 (410/815) [46.8–53.8]
Alcoholism-related conditions (%)	No	89.7 (5360/5977)	43.1 (2310/5360) [41.8–44.4]	44.1 (2365/5360) [42.8–45.5]
	Yes	10.3 (617/5977)	32.6 (201/617) [28.9–36.4]	31.6 (195/617) [28.0–35.4]
Admission at entry (%)	Directly to the medical ED	59.2 (3536/5977)	48.4 (1712/3536) [46.8–50.1]	49.0 (1734/3536) [47.4–50.7]
	General open ED	40.8 (2441/5977)	32.7 (799/2441) [30.9–34.6]	33.8 (826/2441) [32.0–35.8]
Systemic Inflammatory Response Syndrome (SIRS) (%)	No SIRS	61.6 (3681/5977)	24.5 (901/3681) [23.1–25.9]	28.5 (1049/3681) [27.0–30.0]
	SIRS	38.4 (2296/5977)	70.1 (1610/2296) [68.2–72.0]	65.8 (1511/2296) [63.8–67.8]
Number of organ failures (%)	0	57.5 (3434/5977)	32.4 (1113/3434) [30.8–34.0]	34.1 (1170/3434) [32.5–35.7]
	1	29.2 (1745/5977)	49.7 (867/1745) [47.3–52.1]	49.7 (868/1745) [47.4–52.1]
	≥2	13.4 (798/5977)	66.5 (531/798) [63.1–69.8]	65.4 (522/798) [62.0–68.7]

1 95%CI  = 95% confidence intervals.

Of all included patients, 42.0% (N = 2511; 95% confidence interval [95%CI]: 40.8%–43.3%) had one or more community-acquired infections associated with their admission according to chart review, and 42.8% (N = 2560; 95%CI: 41.6%–44.1%) were identified with an infection by discharge diagnoses (Table 1). Of all patients included, 37.1% (N = 2218, 95%CI: 35.9%–38.5%) had one specific site of infection according to chart review, compared to 38.5% (N = 2302, 95%CI: 37.3%–39.8%) identified by discharge diagnoses and 4.9% (N = 293, 95%CI: 4.4%–5.5%) had two or more specific sites of infection according to the chart review, compared to 4.3% (N = 258, 95%CI: 3.8%–4.9%) identified by discharge diagnoses.

**Figure 2:**
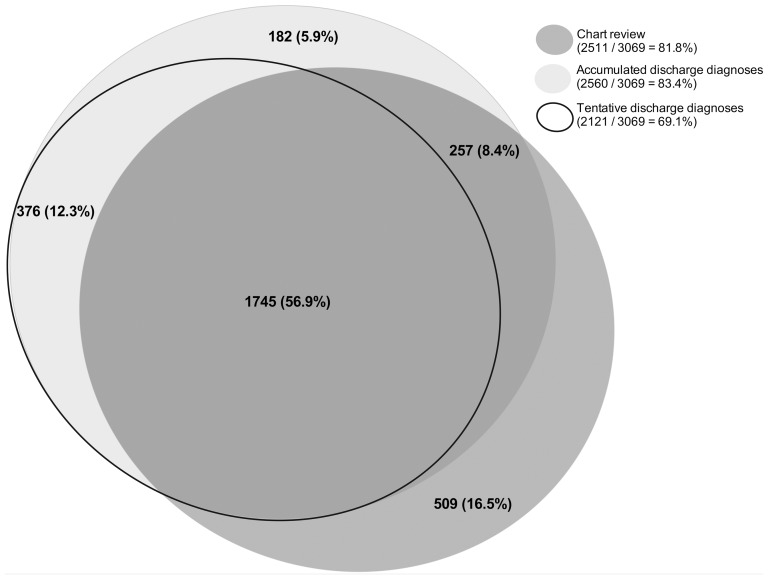
Area proportional Euler Diagram of patients with a diagnosis of infection identified by chart review (dark gray fill), tentative ICD-10 discharge diagnoses from the medical ED (no fill) or accumulated ICD-10 discharge diagnoses from the entire course of admission (light gray fill).

The relations between the diagnosis of infection based on chart review, tentative discharge diagnoses and accumulated discharge diagnoses from the medical emergency department are presented in Figure 2. In total, 3069 patients were identified with an infection by either manual chart review, tentative ICD-10 discharge diagnoses or accumulated ICD-10 discharge diagnoses. The figure shows that 182 (5.9%) patients were registered with a discharge diagnosis of infection after transfer from the medical emergency department to another department at the hospital.

### Number and proportion of patients with infection

**Figure 3:**
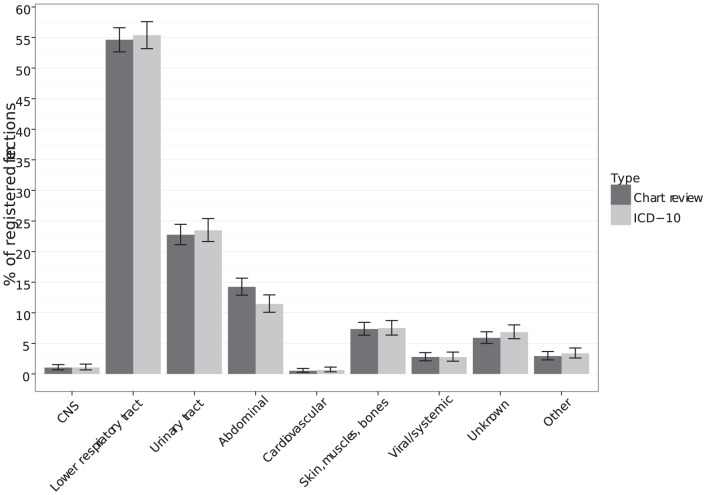
Distribution of sites of infection identified by chart review and by accumulated ICD-10 discharge diagnoses. The prevalence rate could exceed 100%, because a patient could have more than one site of infection per admission. CNS  =  Central nervous system.

The most common site of infection was the lower respiratory tract (with- and without pneumonia) with about 54% of all registered sites of infection, followed by the urinary tract with 22% and abdomen with 14% (Figure 3). We found no difference in numbers of patients with different sites of infection, identified by one or the other method.

When stratifying on patient baseline characteristics, chart review and ICD-10 code, identification of infected patients showed no difference in number and proportion identified (Table 1).

### Identification of individuals with infection

The sensitivity of identifying patients with a community-acquired infection by discharge diagnoses in a medical ED was 79.9% (95%CI: 78.1%–81.3%), specificity of 83.9% (95%CI: 82.6%–85.1%), positive likelihood ratio of 4.95 (95%CI: 4.58–5.36), negative likelihood ratio of 0.24 (95%CI: 0.22–0.26), positive predictive value of 78.2% (95%CI: 76.6%–79.9%) and negative predictive value of 85.1% (95%CI: 83.9%–86.3%) (Table 2).

**Table 2:** Estimation of sensitivity, specificity, predictive values and likelihood ratios of the diagnosis codes of infection in patients admitted to the medical emergency department with- and without infection.

ICD-10 Discharge diagnoses	Chart review (Gold standard)		
	Infection	No infection	Total
Infection	2002	558	2560
No infection	509	2908	3417
Total	2511	3466	5977

	Results (95% confidence intervals)
Sensitivity	79.9% (78.1%–81.3%)
Specificity	83.9% (82.6%–85.1%)
Positive likelihood ratio	4.95 (4.58–5.36)
Negative likelihood ratio	0.24 (0.22–0.26)
Positive predictive value	78.2% (76.6–79.9)
Negative predictive value	85.1% (83.9–86.3)

Although the sensitivity of identifying patients with community-acquired infection by discharge diagnoses increased with old age and number of organ failures, the corresponding positive likelihood ratios decreased due to a decreasing specificity. The demographic characteristic with the highest positive likelihood ratio associated was patients aged 15–39 years (9.8, 95%CI: 7.7–12.5) whereas patients with ≥2 organ failures associated to the admission expressed the lowest positive likelihood ratio (3.0, 95%CI: 2.5–3.7) (Table 3).

**Table 3:** Sensitivity, Specificity, predictive values and likelihood ratios for demographic characteristics in patients admitted with a diagnosis of community-acquired infection.

		Sensitivity % (95%CI)^1^	Specificity % (95%CI)^1^	Positive predictive value % (95%CI)^1^	Negative predictive value % (95%CI)^1^	Positive likelihood ratio (95%CI)^1^	Negative likelihood ratio (95%CI)^1^
Gender	Female	78.7 (76.4–80.8)	82.6 (80.8–84.3)	75.8 (73.4–78.0)	84.8 (83.1–86.4)	4.5 (4.1–5.0)	0.26 (0.23–0.29)
	Male	80.9 (78.6–83.1)	85.5 (83.7–87.3)	81.1 (78.7–83.3)	85.4 (83.6–87.2)	5.6 (4.9–6.3)	0.22 (0.20–0.25)
Age-categories, years	15–39	76.7 (72.0–80.9)	92.2 (90.1–93.9)	81.9 (77.5–85.8)	89.5 (87.2–91.5)	9.8 (7.7–12.5)	0.25 (0.21–0.30)
	40–64	79.7 (76.4–82.8)	88.3 (86.3–90.2)	80.0 (76.7–83.1)	88.2 (86.1–90.0)	6.8 (5.8–8.1)	0.23 (0.20–0.27)
	65–84	79.5 (77.0–81.9)	78.0 (75.5–80.4)	77.4 (74.8–79.8)	80.1 (77.6–82.4)	3.6 (3.2–4.0)	0.26 (0.23–0.30)
	85+	83.1 (79.1–86.6)	72.9 (68.4–77.1)	74.8 (70.6–78.8)	81.6 (77.4–85.4)	3.1 (2.6–3.6)	0.23 (0.19–0.29)
Charlson Comorbidity index	0	81.2 (78.6–83.7)	88.0 (86.3–89.5)	79.1 (76.4–81.6)	89.3 (87.7–90.7)	6.8 (5.9–7.7)	0.21 (0.19–0.24)
	1–2	78.5 (74.9–81.8)	82.5 (79.6–85.2)	77.3 (73.7–80.6)	83.5 (80.7–86.1)	4.5 (3.8–5.3)	0.26 (0.22–0.31)
	>2	79.0 (76.3–81.5)	78.1 (75.5–80.7)	77.8 (75.1–80.4)	79.3 (76.7–81.8)	3.6 (3.2–4.1)	0.27 (0.24–0.30)
Immunosupression	No	80.6 (78.8–82.3)	84.3 (82.9–85.5)	77.3 (75.5–79.1)	86.7 (85.5–87.9)	5.1 (4.7–5.6)	0.23 (0.21–0.25)
	Yes	75.7 (71.5–79.6)	80.9 (76.5–84.8)	82.9 (78.9–86.4)	73.1 (68.5–77.3)	4.0 (3.2–4.9)	0.30 (0.25–0.36)
Alcoholism-related conditions	No	80.3 (78.6–81.9)	83.2 (81.9–84.6)	78.4 (76.7–80.0)	84.8 (83.4–86.0)	4.8 (4.4–5.2)	0.24 (0.22–0.26)
	Yes	73.6 (67.0–79.6)	88.7 (85.3–91.6)	75.9 (69.3–81.7)	87.4 (83.9–90.4)	6.5 (4.9–8.6)	0.30 (0.24–0.38)
Admission at entry	Directly to the Medical ED	80.7 (78.7–82.5)	80.6 (78.8–82.4)	79.6 (77.7–81.5)	81.6 (79.8–83.4)	4.2 (3.8–4.6)	0.24 (0.22–0.26)
	General open ED	77.7 (74.7–80.6)	87.5 (85.8–89.1)	75.2 (72.1–78.1)	89.0 (87.3–90.5)	6.2 (5.4–7.1)	0.25 (0.22–0.29)
Systemic Inflammatory Response Syndrome (SIRS)	No SIRS	73.0 (70.0–75.9)	85.9 (84.6–87.2)	62.7 (59.7–65.7)	90.8 (89.6–91.8)	5.2 (4.7–5.7)	0.31 (0.28–0.35)
	SIRS	83.5 (81.6–85.3)	75.7 (72.3–78.8)	88.9 (87.3–90.5)	66.1 (62.7–69.4)	3.4 (3.0–3.9)	0.22 (0.19–0.25)
Number of organ failures	0	77.4 (74.9–79.9)	86.7 (85.3–88.1)	73.7 (71.1–76.2)	88.9 (87.5–90.2)	5.8 (5.2–6.5)	0.26 (0.23–0.29)
	1	79.8 (77.0–82.4)	80.0 (77.2–82.6)	79.7 (76.9–82.4)	80.0 (77.2–82.6)	4.0 (3.5–4.6)	0.25 (0.22–0.29)
	≥2	84.4 (81.0–87.4)	72.3 (66.5–77.6)	85.8 (82.5–88.7)	69.9 (64.1–75.3)	3.0 (2.5–3.7)	0.22 (0.18–0.27)

1 95%CI  = 95% confidence intervals.

Gold standard manual is chart review.

The discharge diagnoses indicating infection from the most prevalent sites of infection, the lower respiratory tract and urinary tract, showed sensitivities of 70.6% (95%CI: 68.1%–73.0%) and 61.4% (95%CI: 57.3%–65.4%) and positive likelihood ratios of 8.3 (95%CI: 7.5–9.2) and 11.3 (95%CI: 10.0–12.9), respectively (Table 4).

**Table 4:** Sensitivity, Specificity predictive values and likelihood ratios for sites of infection.

Site_of_infection	N, chart review	Sensitivity % (95%CI)^1^	Specificity % (95%CI)^1^	Positive predictive value % (95%CI)^1^	Negative predictive value % (95%CI)^1^	Positive likelihood ratio (95%CI)^1^	Negative likelihood ratio (95%CI)^1^
Central nervous system	26	69.2 (48.2–85.7)	99.8 (99.7–99.9)	64.3 (44.1–81.4)	99.9 (99.7–99.9)	412.0 (210.8–805.3)	0.31 (0.17–0.55)
Lower respiratory tract	1368	70.6 (68.1–73.0)	91.5 (90.6–92.3)	71.1 (68.6–73.5)	91.3 (90.4–92.1)	8.3 (7.5–9.2)	0.32 (0.30–0.35)
Urinary tract	570	61.4 (57.3–65.4)	94.6 (93.9–95.2)	54.4 (50.5–58.3)	95.9 (95.3–96.4)	11.3 (10.0–12.9)	0.41 (0.37–0.45)
Abdominal	357	57.1 (51.8–62.3)	98.6 (98.3–98.9)	72.6 (67.0–77.7)	97.3 (96.9–97.7)	41.7 (32.8–53.0)	0.43 (0.39–0.49)
Cardiovascular	13	84.6 (54.6–98.1)	99.9 (99.8–100.0)	73.3 (44.9–92.2)	100.0 (99.9–100.0)	1261.6 (461.0–3452.5)	0.15 (0.04–0.55)
Skin, muscles, bones	183	69.4 (62.2–76.0)	99.4 (99.2–99.6)	79.4 (72.3–85.4)	99.0 (98.8–99.3)	121.8 (85.6–173.5)	0.31 (0.25–0.38)
Viral/systemic	69	53.6 (41.2–65.7)	99.5 (99.3–99.7)	57.8 (44.8–70.1)	99.5 (99.2–99.6)	117.3 (75.9–181.4)	0.47 (0.36–0.60)
Unknown	149	34.2 (26.7–42.4)	97.4 (97.0–97.8)	25.5 (19.6–32.1)	98.3 (97.9–98.6)	13.4 (10.2–17.6)	0.67 (0.60–0.76)
Other sites of infection	74	54.1 (42.1–65.7)	99.2 (99.0–99.4)	47.1 (36.1–58.2)	99.4 (99.2–99.6)	70.9 (49.5–101.5)	0.46 (0.36–0.59)
No infection	3471	81.8 (80.5–83.1)	80.6 (79.0–82.1)	85.4 (84.1–86.6)	76.2 (74.5–77.8)	4.2 (3.9–4.6)	0.23 (0.21–0.24)

1 95%CI  = 95% confidence intervals.

The added number of individual sites of infections exceed the number of patients with infection, because one patient could have more than one site of infection.

## Discussion

Our study explored the possibility of using ICD-10 discharge diagnoses from health administrative data to identify patients with infections presenting at a medical ED. We found, that using discharge diagnoses as surrogate markers of infection gave reliable estimates of numbers and proportions as well as a high degree of validity when stratifying on the different sites of infection, although it only had moderate capability to identify patients with infections in general.

We found that over 40% of all patients presented to the medical ED with a community-acquired infection. The most common site of infection was the lower respiratory tract, which concurs with prior studies [15]–[19]. The cohort of infected patients identified by discharge diagnoses, and that identified by chart review, were almost similar with regards to the prevalence rate of infection, distribution of patient characteristics, sites of infection and disease severity.

We chose to use the CDC/NHSN criteria as gold standard in our chart review. Since the criteria primarily were developed to survey healthcare-acquired infections, we had to adapt them to community-acquired infections. Some patients had clinically evident infections that did not conform to the CDC/NHSN defined criteria, so we used an alternative predefined definition in these cases, described in S 1. Use of another gold standard might have resulted in different results, but the CDC/NHSN criteria was chosen because of its widespread use the last 25 years [6]. A recent study assessed the inter-observer agreement of CDC/NHSN for classifying infections in critically ill patients in an ICU setting and found excellent agreement, but also found that full concordance on all aspects of the diagnosis of a specific infection was rare[20].

We found, that patients with one or more discharge diagnoses of infection, accumulated throughout their entire course of admission, were 4.9 times more likely to have a community-acquired infection, compared to patients without any confirmed infection. While a positive likelihood ratio greater than 10 means a test is good at ruling in a diagnosis [21], our results indicate, that discharge diagnoses as surrogate markers of infection are less good as an identification method of patients admitted to a medical ED with diagnoses of infections in general.

Only few studies have validated the use of hospital administrative data as a surrogate marker of infections in general. These studies, as the present study, yielded moderate to high positive predictive values (54–90%) [2]–[5]. When restricting the diagnoses to specific sites of infection, we found increased and acceptable high positive likelihood ratios and a high degree of validity in almost every site except the lower respiratory tract. This could be due to the classification criteria we used or to a non-specific coding practice in this patient group. Prior studies show similar results of low sensitivity and positive likelihood ratios in diagnosing pneumonia by discharge diagnoses in different patient populations [22]–[24], but these studies did not include lower respiratory tract infections without pneumonia as we did in the present study. In a sub-group analysis, where we divided the lower respiratory tract infections into pneumonia and lower respiratory tract infections without pneumonia, we found a slight decrease in positive likelihood ratios in patients with pneumonia, compared to the combined group of lower respiratory tract infections (data not shown).

If the administrative data is used to identify patients with infection for predictive analysis, it is not only important that the administrative data identify patients with infections and rule out patients without infections, but also that the measures of validity remain constant within patient sub-groups. We found a decreasing positive likelihood ratio with increasing disease severity, older age, severe comorbidity and presence of immunosuppression. These findings indicate a high degree of differential misclassification; hence the discharge diagnoses lead to an overrepresentation of young patients without organ dysfunction and morbidity, compared to results identified by manual chart review. This information is important when planning prognostic studies using administrative data to identify patients with infection.

The observed trend in likelihood ratios could be due to a more complex clinical presentation, which older and more comorbid patients present with to the emergency department, making it more difficult to distinguish signs and symptoms of an infection from underlying diseases. Søgaard et al showed similar results in terms of decreasing positive predictive values when validating ICD-10 discharge diagnoses of pleural empyema [25], where younger patients had higher positive predictive values than older patients.

### Strengths and limitations

The strength of the study is the large and unselected cohort of acute medical patients. Several studies have assessed the validity of distinct infections by identifying patients from discharge diagnosis, and subsequently reviewing charts to confirm the diagnosis [3], [25]. This method leaves all patients with an infection, but without a discharge diagnosis of infection, undetected, thus potentially underestimating the “true” prevalence and lacking the ability to report likelihood ratios and sensitivity. Although this study provides information that confirms this method of sampling patients as acceptable when you work with total numbers, trends and proportions as well as site-specific infections it also illustrates the need for careful case validation if ICD-10 identified patients are used in studies assessing risk factors and prognosis.

Due to the uniformly organised Danish public healthcare system we could identify all but three patients included in the study. Another strength is the chart review to identify the reference cohort and subsequent validation of this cohort, yielding a kappa value of 0.67. This shows, that even when a well-defined classification of infection is applied, it is difficult to obtain a high concordance in inter-rater agreement in retrospective studies.

Like some of the prior studies using health administrative data to identify infections, we chose to include all discharge diagnoses from each patient stay as a surrogate marker of infection [19]. Other studies have used tentative admission diagnoses [16] or the primarily assigned discharge diagnoses [26], but because of differences in coding traditions across countries, it is difficult to generalise results and incidence rates. In contrast to the United States, coding of discharge diagnoses in Denmark depends on the treating physician, and a new set of codes might be produced every time the patient is transferred between different departments. As patients from the open general emergency department or the medical ED sometimes are transferred to other departments before a final diagnosis is established, the open general EDs/medical EDs tend to produce more non-specific symptom related codes [27]. Despite differences in coding practices, we found results of positive predictive values comparable with studies conducted in the United States [2]–[5].

It is possible, that we identified hospital-acquired infections in the accumulated discharge diagnoses. However, only 5.9% of all patients had discharge diagnoses of infections added after being transferred from the medical ED to another department, indicating either a very low infection rate or insufficient coding practice of hospital-acquired infections. The current work was a single-center study from a medical ED at a university hospital; therefore, the results of this study may not be generalisable to other hospitals, surgical departments or intensive care units. However the hospital serves as the primary (and only) hospital for all residents in the catchment area, which minimise selection bias and probably increase the generalisability to other primary hospitals. The most appropriate design would have been a multicentre- and maybe international study with a larger sample size.

We chose to use ICD-10 discharge diagnoses of infection based on a review of all discharge diagnoses accumulated throughout the screened patients course of admissions. We based our choice of codes on the adapted ICD-10 version of the ones by Angus et al. from 2001, [26] but found that they were insufficient regarding some of the codes in our study population and we therefore included these clinical relevant missing codes. Another definition of ICD-10 diagnoses identifying infections could possibly have affected the main results.

## Conclusion

Using ICD-10 discharge diagnoses as surrogate markers of infection yield almost the same prevalence rate, distribution of sites of infection and distribution of demographic characteristics compared to chart review. Identifying patients with site-specific infections showed a high degree of validity, but only moderate validity when identifying infections in general.

## Supporting Information

Appendix S1 Definition of infections identified by chart review.
(DOCX)

Appendix S2 ICD-10 codes identifying infections.
(DOCX)

Appendix S3 Definition of covariates in baseline characteristics.
(DOCX)
