# Supplementary material for: How Well Do Discharge Diagnoses Identify Hospitalised Patients with Community-Acquired Infections? – A Validation Study
Source: PLoS One. 2014 Mar 24;9(3):e92891. doi: 10.1371/journal.pone.0092891 (PMC3963967; doi:10.1371/journal.pone.0092891)
Supplement: Appendix S1 — Definition of infections identified by chart review. (DOCX) [file pone.0092891.s001.docx]

**Appendix S1: Definition of infections in chart review**

Signs, symptoms and laboratory values were evaluated the first 24 hours after arrival to the hospital

Diagnostic tests and microbiological samples were evaluated the first 48 hours after arrival with the exception of radiographic sign of ostitis/osteomyelitis, where time frame was 5 days.

**Adapted CDC/NHSN surveillance definition of health care–associated infection and criteria for specific types of infections in the acute care setting**(Horan, Andrus, & Dudeck, 2008)**. Only the changes from the original criteria are listed below.**

The criteria marked with * are changes to the original CDC/NHSN criteria

**2: PULMONARY**

*Other infections of the lower respiratory tract*

Other infections of the lower respiratory tract must meet at least 1 of the following criteria:

1. Patient has organisms seen on smear or cultured from lung tissue or fluid, including pleural fluid.

2. Patient has a lung abscess or empyema seen during a surgical operation or histopathologic examination.

3. Patient has an abscess cavity seen on radiographic examination of lung.

*4. Patient has purulent pleural fluid

*Pneumonia*

1: At least one of the following X-ray or CT scan, new or progressive:

a. Infiltrate

b. Consolidation

c. Cavitation

AND at least one of the following signs:

a. Fever (>38C) with no other cause

b. Leukopenia (<4,000 WBC/mm3) OR leukocytosis (≥ 12,000 WBC/mm3)

c. Altered mental status with no other cause, in ≥ 70 y.o.

AND at least two of the following symptoms:

a. New onset of purulent sputum (*yellow, brown or green coloured) OR change in character of sputum OR increased respiratory secretions OR increased suctioning requirements

b. New onset or worsening cough OR dyspnea (a subjective complaint from the patient registered in the electronic patient journal) OR tachypnea (respiratory rate>25)

c. Rales OR broncial breath sounds

d. Worsening gas exchange: O2 desat (*<90% in patients with known COPD OR <94% in patients without COPD) OR increased O2 requirements OR increased ventilation demand

**Lower respiratory tract infection without pneumonia*

*Same criteria as pneumonia but without an X-ray or CT scan indicating a new or progressive infiltrate, consolidation or cavitation

**3: URINARY TRACT INFECTION**

*Asymptomatic bacteriuria**We did not identify asymptomatic bacteriuria in the manual chart review

**9: OTHER SITES OF INFECTION**

**EYE, EAR, NOSE, THROAT, OR MOUTH INFECTION**

*Conjunctivitis*

*We did not identify patients with conjunctivitis in the manual chart review

**Alternative definitions of infections in patients with a clinical evident infection, but not conforming to the CDC/NHSN definition:**

Infection was defined as identification of a relevant pathogen by microscopy/culture/polymerase chain reaction, positive serology, pneumonia verified by chest X-ray, infection documented with other imaging techniques, positive urine dip test combined with symptoms of urine tract infection, or as typical clinical symptoms such as erysipelas.

**References**

Horan, T. C., Andrus, M., & Dudeck, M. a. (2008). CDC/NHSN surveillance definition of health care-associated infection and criteria for specific types of infections in the acute care setting. *American Journal of Infection Control*, *36*(5), 309–32. doi:10.1016/j.ajic.2008.03.002
