# Supplementary material for: How Well Do Discharge Diagnoses Identify Hospitalised Patients with Community-Acquired Infections? – A Validation Study
Source: PLoS One. 2014 Mar 24;9(3):e92891. doi: 10.1371/journal.pone.0092891 (PMC3963967; doi:10.1371/journal.pone.0092891)
Supplement: Appendix S2 — ICD-10 codes identifying infections. (DOCX) [file pone.0092891.s002.docx]

**Appendix S2: ICD-10 codes identifying infections**.

| Site of infection | ICD-10 discharge diagnosis |
| --- | --- |
| Central nervous system | A39.0, A39.2A, A86.9, A87.0, A87.9, B00.3, B00.4, B02.0, B02.2, B02.2A, B02.2B, B91.9, G00.1, G00.8, G00.9, G00.9A, G01.9, G04.0, G04.2, G06.0, G06.0F, G06.2, G07.9 |
|  |  |
| Lower respiratory tract | Pneumonia: A31.0A, A48.1, B37.1, J12.0, J13.9, J14.9, J15,J15.0, J15.1, J15.2, J15.4, J15.5, J15.7, J15.8, J15.9, J17.0, J17.8C, J18, J18.0, J18.1, J18.8, J18.9, J20.9, J20.9A, J21.9, J22.9, J69.0, J69.8, J69.8A  Other: A15.0, A15.1, A15.2, A15.9, B90.9, J40.9, J44.0, J85.1, J85.2, J86.0, J86.9 |
|  |  |
| Urinary tract | A41.9B, N10.9, N12.9, N13.6, N30.0, N30.8, N30.9, N39.0, N39.0B |
|  |  |
| Abdominal | A00.9, A01.1, A02.0, A03.8, A04.3, A04.5, A04.7, A04.8, A04.9, A05.9, A08.0, A08.1, A08.2, A08.3, A08.4, A08.5, A09, A09.9, B67.0, K35.0, K35.0A, K35.1, K35.1A, K35.9, K57.2B, K57.3, K57.3A, K57.3B, K57.3F, K57.9A, K65.0,K65.0A, K65.0G, K65.0J, K65.8, K65.8I, K65.9, K75.0, K80.3, K80.4, K81.0, K81.9, K83.0 |
|  |  |
| Cardiovascular | I30.0, I30.1, I30.8, I30.9, I33.0, I33.9, I38.9, I39.8 |
|  |  |
| Skin, muscles, bones | A46.9, B00.1A,B00.1B, B37.2, K61.0, K61.0A, K61.1, K61.2, L02.2, L02.2T, L02.4, L02.4F, L02.4K, L02.9, L02.9A, L03.1, L03.1E, L03.3, L08.8, L08.9, M00.0, M00.2, M00.2A, M00.8, M00.9, M46.3, M46.4, M46.5, M46.5A, M46.9, M71.1, M86.1, M86.8, M86.9 |
|  |  |
| Viral/systemic | A51.5, A79.9, B00.1, B05.9, B20.4, B20.6, B20.8, B23.0, B23.2, B24.9, B25.8, B25.9, B27.0, B27.9, B50.9, B52.9, B54.9, B55.0, B58.9, J09.1, J09.9, J10.0, J10.8, J11, J11.0, J11.1, J11.8 |
|  |  |
| Unknown | A40.1, A40.3, A40.8, A40.9, A41.0, A41.1, A41.1A, A41.2, A41.3, A41.4, A41.5, A41.8, A41.9, A49.9A, B37.7, A32.9, A41.9A, A42.9, A44.9, A48.2, A49.0, A49.1, A49.3, A49.8, A49.9, A68.9, A70.9, A81.2, B00.8, B02.9, B34.0, B34.9, B36.9, B37.0, B37.8, B80.9, B89.9, B95.5, B95.6, B95.6A, B96.4, B96.5, B96.8, B99.9, R50, R50.0, R50.8, R50.9, T81.4D, T84.6, T89.9 |
|  |  |
| Other sites of infection | B00.2A, B02.3G, B37.3A, B37.4, B37.8C, E06.0, E06.1, H65.1, H66.0, H66.9, J00.9B, J01.0, J01.1, J01.2, J01.8, J01.9, J02.0, J02.9, J02.9B, J03.0, J03.9, J03.9A, J04.0, J05.1, J06.9, J36.9, J39.0C, K04.0A, K05.3A, K10.2C, K11.2C, K12.1, K62.8L, N41.2, N45.0B, N45.9, N45.9A, N76.4A, O86.8 |
|  |  |
