# Supplementary material for: How Well Do Discharge Diagnoses Identify Hospitalised Patients with Community-Acquired Infections? – A Validation Study
Source: PLoS One. 2014 Mar 24;9(3):e92891. doi: 10.1371/journal.pone.0092891 (PMC3963967; doi:10.1371/journal.pone.0092891)
Supplement: Appendix S3 — Definition of covariates in baseline characteristics. (DOCX) [file pone.0092891.s003.docx]

**Appendix S3: Definition of covariates in baseline characteristics**

**Immunosuppression was defined as one or more of the following before current admission:**1: A moderate to high intake of immunosuppressant medication (ATC: H02AB* or L00-L04, cumulative defined daily dose ≥ 120 in the preceding 120 days with a compliance rate of 80%)
2: Primary immunodeficiency diseases (ICD-10: D61; D70-D71; D80-D82; D84; E70.3; G11.3)
3: Acquired Immune Deficiency Syndrome defining diseases (ICD-10: B20 – B24; F02.41- F02.44)
4: A newly diagnosed malignancy registered in the Danish Cancer Registry, up to a year prior admission ICD-10: C00-C79; C97; D37-D44, D48)
5: Diagnosis of organ transplantation (ICD-10: Z94)

**Alcoholism-related condition was defined as at least one of the following before the current admission:**1: A redeemed prescription of Disulfiram (ATC: N07BB01) from 2007 and onwards
2: ≥2 admissions with a discharge diagnosis of an acute alcohol episode (ICD-10: F10.0-F10.1; T519)
3: At least one admission with a discharge diagnosis of a chronic alcohol related diagnosis (ICD-10: F10.2 -F10.9; E24.4; E52.9A; G31.2; G62.1; G72.1; I42.6; K29.2; K70; K85.2; K86.0; L27.8A; O35.4; P04.3; T50.0A; Z71.4; Z72.1; Z50.2)
4: A registration in The National Register of Alcohol Abuse Treatment.

**Community-acquired bacteraemia** was defined, as having a positive blood culture drawn within the first two days of admission. A blood culture consisted of two blood culture sets (each comprising one aerobic and one anaerobic bottle) and we defined bacteraemia as either: (1) recognised pathogens detected in ≥1 blood culture, or (2) common skin contaminants (coagulase-negative staphylococci, *Bacillus* spp, *Propionibacterium* spp, *Corynebacterium* spp, viridans group streptococci, *Aerococcus* spp, or *Micrococcus* spp) detected in ≥2 blood culture sets within 5 days. The date of the first positive blood culture set was regarded as the date of bacteraemia. Polymicrobial bacteraemia was defined as isolation of ≥2 different microorganisms, deemed to represent bacteraemia, within 2 days. Microbiological methods

The blood cultures were incubated and screened for growth of microorganisms for 6 days or until detected positive, using the Bactec 9240 system (Becton Dickinson, NJ, USA) until January 2011 and the Bact/Alert system (BioMérieux) thereafter. Routine methods for identification of bacteria were based on conventional characterisation [[14](javascript:parent.onLocalLink('_ENREF_14',window.frameElement))], the Danish reference programme (http://www.dskm.dk), and automated identification using Vitek 2 (bioMérieux) and MALDI-TOF (SARAMIS, bioMérieux).
